# Binding and dimerization of PGLa peptides in anionic lipid bilayer studied by replica exchange molecular dynamics

**DOI:** 10.1038/s41598-024-55270-8

**Published:** 2024-02-29

**Authors:** Steven R. Bowers, Christopher Lockhart, Dmitri K. Klimov

**Affiliations:** School of Systems Biology, George Mason University, Manassas, VA 20110 USA

**Keywords:** Protein aggregation, Molecular modelling, Membrane structure and assembly

## Abstract

The 21-residue PGLa peptide is well known for antimicrobial activity attributed to its ability to compromize bacterial membranes. Using all-atom explicit solvent replica exchange molecular dynamics with solute tempering, we studied PGLa binding to a model anionic DMPC/DMPG bilayer at the high peptide:lipid ratio that promotes PGLa dimerization (a two peptides per leaflet system). As a reference we used our previous simulations at the low peptide:lipid ratio (a one peptide per leaflet system). We found that the increase in the peptide:lipid ratio suppresses PGLa helical propensity, tilts the bound peptide toward the bilayer hydrophobic core, and forces it deeper into the bilayer. Surprisingly, at the high peptide:lipid ratio PGLa binding induces weaker bilayer thinning, but deeper water permeation. We explain these effects by the cross-correlations between lipid shells surrounding PGLa that leads to a much diminished efflux of DMPC lipids from the peptide proximity at the high peptide:lipid ratio. Consistent with the experimental data the propensity for PGLa dimerization was found to be weak resulting in coexistence of monomers and dimers with distinctive properties. PGLa dimers assemble via apolar criss-cross interface and become partially expelled from the bilayer residing at the bilayer-water boundary. We rationalize their properties by the dimer tendency to preserve favorable electrostatic interactions between lysine and phosphate lipid groups as well as to avoid electrostatic repulsion between lysines in the low dielectric environment of the bilayer core. PGLa homedimer interface is predicted to be distinct from that involved in PGLa-magainin heterodimers.

## Introduction

Emergence of antibiotic-resistant bacteria has sparked an interest in antimicrobial peptides (AMP), which are seen as a potential new class of antimicrobial agents^1^. AMPs constitute a component of the innate immune system^2^, and, because their mechanism of action relies on recognition of microbial membranes, the development of resistance against them is less likely compared to traditional antibiotics^3^. Indeed, a strong argument in favor of membrane-centric mechanism is provided by the observation that the antimicrobial functions of AMPs are retained by their D-isomeric variants^4^. AMPs present a rich palette of biological functions, including antimicrobial activity, cytotoxicity, cell penetration, and amyloidogenicity. An AMP that has been intensively studied experimentally and computationally is a 21-mer cationic amphipathic peptide PGLa isolated from the skin glands of *Xenopus laevis* frogs^5,6^. PGLa displays strong activity against gram-negative and gram-positive bacteria and fungi^7^. Although the exact cytotoxic mechanism of PGLa and AMPs in general awaits elucidation, the formation of pores in cellular membranes is the oldest suggested mechanism seeking to explain their antimicrobial action^7^. In its support, recent experiments and modeling have shown that, consistent with pore formation, PGLa induces permeation of calcein through DOPC/DOPG bilayers^8,9^. However, to understand PGLa function on a molecular level one needs to study not only its interactions with lipid bilayers but also PGLa conformational ensembles and states adopted within the membranes.

Cationic PGLa peptides exhibit strong affinity to anionic bacteria-like bilayers^10^ and, importantly, due to strong hydrophobic moment they change the secondary structure upon binding. Indeed, circular dichroism data indicated that PGLa in water is unstructured^11^, but upon binding to POPC/POPG 3:1 micelles the PGLa residues 6-21 adopt a helical state^12^. This finding has been later confirmed for zwitterionic DMPC bilayers^13^. Significant experimental efforts has been directed toward elucidation of PGLa position in lipid bilayers. Solid state NMR experiments have defined two PGLa states in the membrane liquid crystalline phase, S- and T-states^14^. At low peptide:lipid ratio (1:200) PGLa adopts a surface bound S-state with almost parallel orientation of the peptide with respect to the DMPC/DMPG bilayer and upward orientation of lysine side chains^15,16^. However, at higher peptide:lipid ratio (1:50) PGLa shifts to a tilted T-state, in which the C-terminus inserts into the bilayer^15–18^. In fact, oriented circular dichroism (CD) spectra provided an estimate of the free energy of PGLa homodimers^19^. PGLa is also well known to form heterogeneous aggregates with another AMP magainin resulting in a synergistic increase in antimicrobial action^20–23^. Although the precise heterodimer structure remains unknown, a combination of experimental techniques suggested that glycine PGLa amino acids Gly7 and Gly11 play a critical role in PGLa-magainin aggregation. It is also of note that PGLa states depend on bilayer curvature^24,25^, thickness of bilayer hydrophobic core^17^, and temperature^26^. Specifically, an increase in temperature induces the PGLa transition from T to S states in the liquid crystalline phase. Binding of PGLa to the DMPC/DMPG bilayer has been shown to lead to significant disordering in lipid structure^27^.

In parallel to experimental investigations, molecular dynamics (MD) simulations have been used to probe PGLa interactions with lipid bilayers. Recent 1 μs long MD simulations based on AMBER force field have revealed the helical secondary structure and orientation, including tilt and side chain rotation angles, of PGLa monomers in the DMPC bilayer to be in good agreement with the NMR analysis^28^. Separate AMBER MD simulations showed that the orientation of L18W-PGLa peptide in POPE/POPG bilayer is consistent with X-ray and neutron scattering data^21^. It followed from the MD simulations utilizing CHARMM force field that PGLa monomers bound to the DMPC membrane maintain helical structure and reside just below the phosphorus lipid atoms^29,30^. The analysis of preconstructed antiparallel PGLa dimers has indicated that such assemblies have higher stability than parallel dimers^29^. In order to probe the conformational ensemble of PGLa peptides, our group has performed replica exchange with solute tempering (REST) simulations of PGLa binding to anionic DMPC/DMPG bilayers^31,32^ at the low peptide:lipid (P:L) ratio. By analyzing the binding free energy landscape we identified two major bound states, a metastable surface bound state and a dominant inserted state. In both states positively charged PGLa amino acids maintain electrostatic interactions with anionic phosphate groups by rotating the PGLa helix around its axis. PGLa binding causes an influx of anionic DMPG and efflux of zwitterionic DMPC lipids from the peptide proximity. The analysis of binding free energy suggested that PGLa binding to the DMPC/DMPG bilayer is governed by the balance between desolvation of PGLa positive charges and formation of electrostatic PGLa-lipid interactions.

In this paper, we extended our REST investigation to the PGLa peptides binding to the DMPC/DMPG bilayer at the high P:L ratio. Computation of free energy landscapes suggests that PGLa homodimers are diverse and metastable existing in dynamic equilibrium with monomeric species. Importantly, the PGLa monomers occurring at the high P:L ratio are distinct from those observed at the low ratio. Unexpectedly, PGLa interpeptide interactions reduces the peptide helical propensity and forces the dimerized peptides to position themselves closer to the bilayer surface adopting a criss-cross dimer interface. We also found that the high P:L ratio results in profoundly weaker efflux of zwitterionic lipids from the PGLa binding footprint. The physicochemical mechanism, which changes the properties of PGLa at the high P:L ratio, is proposed.

## Results and discussion

### PGLa properties and bilayer structure at the high peptide:lipid ratio

**Figure 1:**
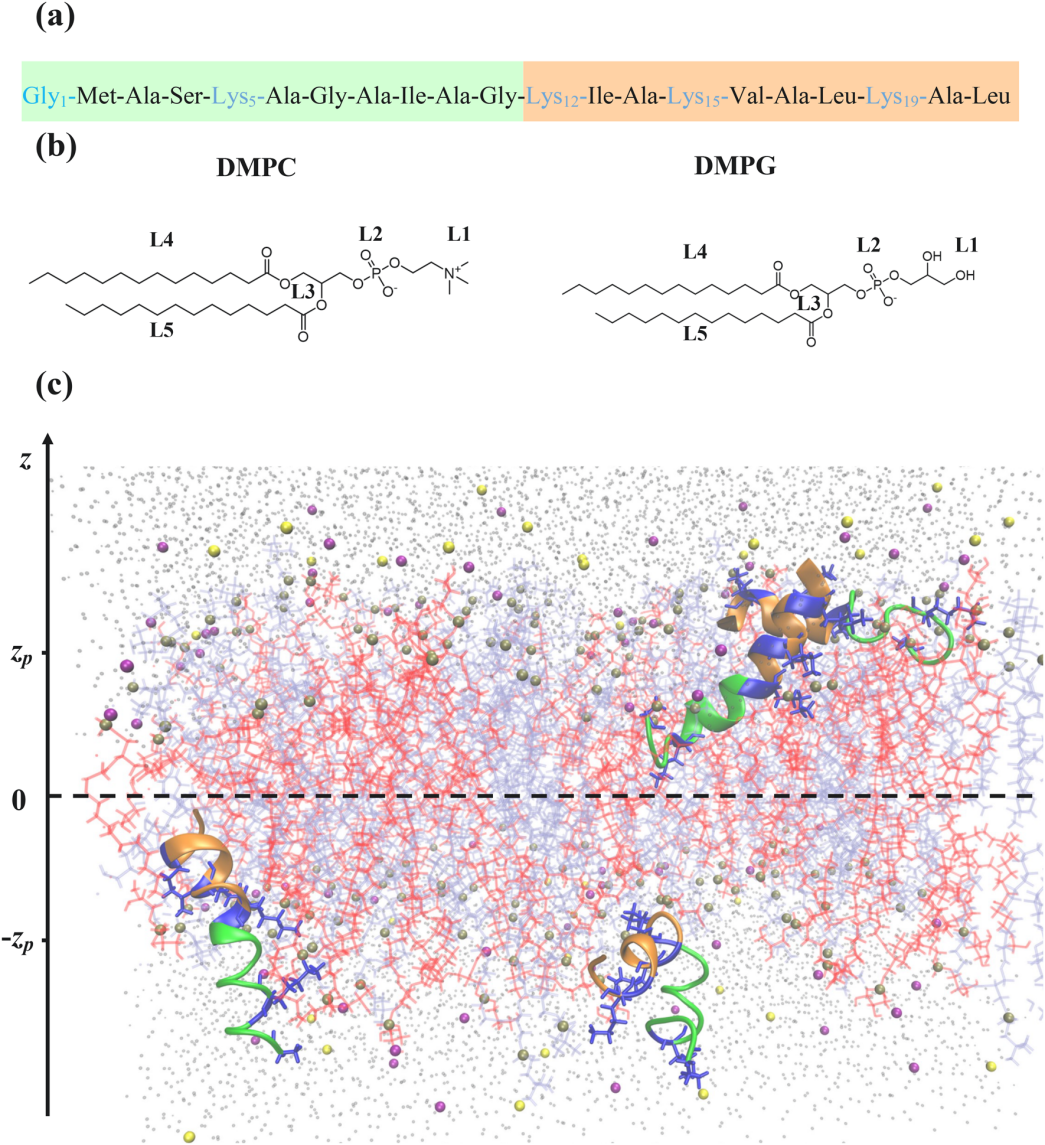
(**a**) PGLa peptide sequence. N- and C-terminal regions are shown in pale green and orange. Positively charged amino acids are in blue. (**b**) Structures of DMPC or DMPG lipids, which are composed of a choline or glycerol group (L1), phosphate group (L2) with phosphorus P atom, glycero backbone (L3), and two fatty acid tails (L4 and L5). L1-L3 represent polar headgroups, whereas L4 and L5 constitute the hydrophobic core. (**c**) A snapshot of REST simulation system. A pair of PGLa peptides bind to each bilayer leaflet. The bilayer is composed of DMPC/DMPG lipids in pale blue and red, respectively. Peptides are colored following (**a**). Water is shown as dots, sodium and chlorine ions are purple and yellow spheres, Lys amino acids are in dark blue, and phosphorus P atoms are in tan. PGLa dimer is formed in the upper leaflet.

*PGLa properties within the DMPG/DMPC bilayer:* Our REST simulations examined PGLa peptides binding to the anionic DMPC/DMPG (3:2) bilayer at the peptide:lipid (P:L) ratio of $\sim$ 1:25 (Fig. 1). At 330 K PGLa binds to the bilayer and dimerizes with the probabilities $P_b\sim 1.0$ and $P_d=0.29\pm 0.05$, respectively. Therefore, the simulations produced the conformational ensemble composed of a mixture of bound PGLa monomers and dimers. Because multiple PGLa species, monomers or dimers, coexist, we refer to them collectively as those sampled at the high P:L ratio. As a reference we use the PGLa binding to the same bilayer at 310 K and lower P:L ratio 1:50 reported previously^32^. The use of “high” and “low” P:L ratio terms for the simulations requires caution. Due to simulation design at the low P:L ratio PGLa cannot aggregate, whereas at the high P:L ratio the peptide aggregation is limited to dimers. Therefore, an alternative reference to the high and low P:L ratio simulations could be 2- or 1-peptide systems, respectively. We discuss the correspondence between the experimental and computational P:L ratios later in this section. Also, in Supplementary Information (SI) we show that the temperature dependence of PGLa properties in CHARMM force field is weak. This circumstance allows us to compare the PGLa properties observed at the high P:L ratio and 330K with those at the low P:L ratio and 310K.

**Figure 2:**
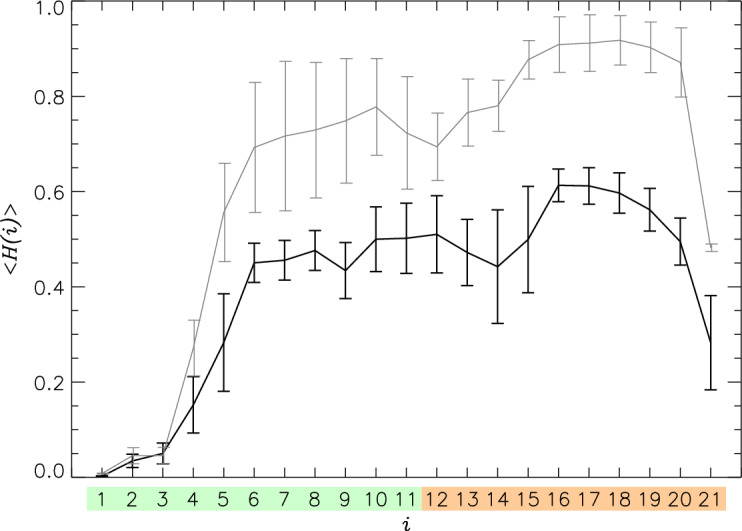
The helical $\langle H(i) \rangle$ fractions are computed for PGLa amino acids *i*. Data in black and grey represent the high and low^32^ P:L ratios. Sampling errors are given by vertical bars. The amino acids from Nt and Ct regions are colored following Fig. 1a. The figure shows that the high P:L ratio suppresses helical structure.

Figure 2 shows that at the high P:L ratio the helical propensity $\langle H(i) \rangle$ is reduced for all PGLa amino acids. Indeed, the overall helical fraction $\langle H \rangle$ is $0.40\pm 0.06$ at the high P:L ratio compared to $0.63\pm 0.05$ at the low, i.e., it is reduced by more than a third. Furthermore, at the low P:L ratio 17 PGLa amino acids are classified as helical for their $\langle H(i) \rangle >0.5$, whereas at the high ratio their number decreases three-fold to 6. The helical propensity is particularly suppressed in the C-terminus, where $\langle H(Ct) \rangle$ reaches $0.81\pm 0.05$ at the low P:L ratio, but drops to $0.51\pm 0.07$ at the high ratio. Besides helix at the high P:L ratio, PGLa forms turns and random coil with their fractions being $0.49\pm 0.06$ and $0.10\pm 0.02$. Multiple experiments have reported the formation of helix in PGLa. According to NMR data probing PGLa binding to 3:1 POPC/POPG micelles at the low 1:100 P:L ratio, the peptide is mostly helical from the position $i=6$ to 21^12^. Similarly, CD studies showed that upon binding to DMPC/DMPG bilayer the PGLa helical fraction *H* varies from 0.67 to 0.76 in the temperature range from 303 to 318K at the P:L ratio of 1:200^10^. A more recent CD study of a similar system at the P:L ratios of 1:200 and 1:50 reported *H* to be about 0.6^14^. These findings are in good agreement with our simulations of PGLa monomer^32^. Raman spectroscopy experiments reported PGLa secondary structure upon binding to DMPC liposomes at much higher P:L ratio of about 1:3 and ambient temperature^33^. In contrast to the data at lower P:L ratios, the PGLa helical structure propensity was strikigly lower reduced to $0.42\pm 0.05$. In qualitative agreement, our simulations report that at the elevated P:L ratio $<H>$ dropped to $0.40\pm 0.06$. There are no experimental data on the helical fraction at the P:L ratio exceeding 1:50. Thus, it appears that the PGLa helix structure is sensitive to P:L ratio and partially unravels with its increase. We have developed a rationale for decreasing helical fraction with the increase in P:L ratio, which is proposed after the analysis of lipid distributions. Because it is challenging to unambiguously match the experimental and simulation P:L ratios (see below), our prediction that $<H>$ decreases with the P:L ratio should be viewed as preliminary, and our simulations may still underestimate $<H>$.

**Figure 3:**
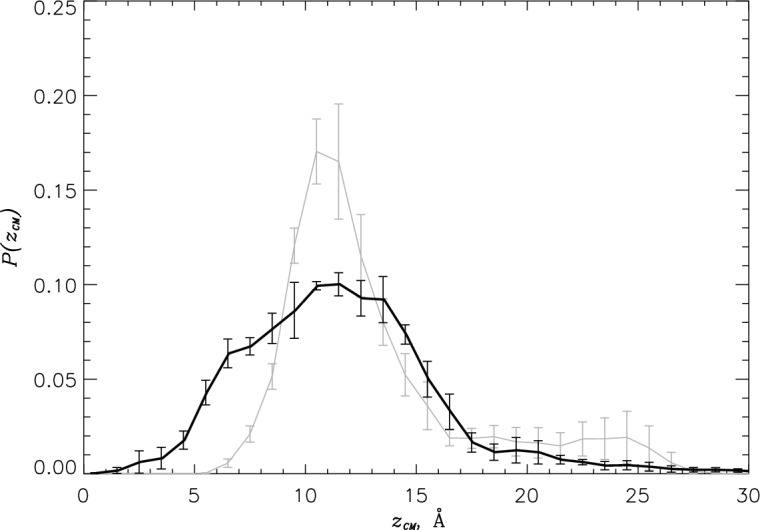
The probability distributions $P(z_{CM})$ report the location of the PGLa center of mass $z_{CM}$ along the bilayer normal. Data in black and grey correspond to the high and low^32^ P:L ratios. The figure reveals deeper insertion of PGLa peptides at the high P:L ratio.

To map the PGLa location within the DMPC/DMPG bilayer, we first computed the probability distribution $P(z_{CM})$ of the PGLa peptide center of mass $z_{CM}$ along the bilayer normal. Figure 3 reveals that with the P:L ratio increase the peptide shifts deeper into the bilayer. In fact, the average position of the PGLa center of mass $\langle z_{CM} \rangle$ changes from $13.1\pm 0.9$ at the low to $11.6\pm 0.5$ Å at the high P:L ratio. To get further insight, we used the probabilities *P*(*z*; *i*) for amino acid *i* to occur at the distance *z* from the bilayer midplane to compute the average positions of amino acids $\langle z(i) \rangle$ (see Methods). We found that with the increase in P:L ratio there is a discernible deeper insertion of PGLa C-terminal amino acids, and at both P:L ratios all amino acids, on an average, reside below the position of the center of mass of phosphorus atoms in a leaflet $z_P$. Thus, at both P:L ratios all PGLa amino acids are classified as inserted (see Methods). Previous microsecond-long simulations, which did not utilize enhanced sampling, also found that PGLa monomers are deeply inserted in the DMPC bilayer residing well below phosphate groups with their center of mass $z_{CM}=9.4$ Å^29^.

**Figure 4:**
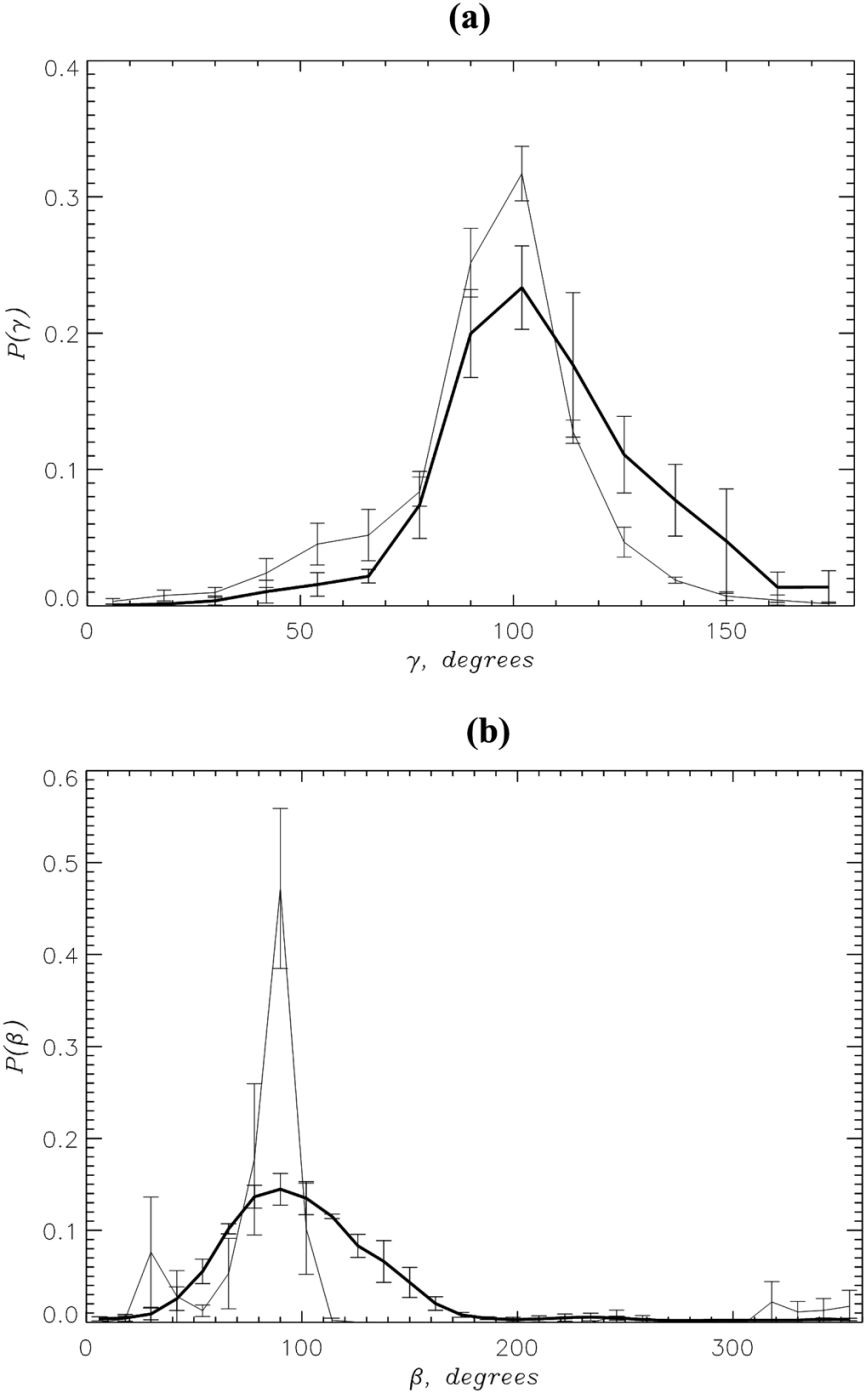
(**a**) The probability distributions $P(\gamma )$ probe the PGLa peptide tilt within the lipid bilayer. (**b**) The probability distributions $P(\beta )$ describe the orientation of PGLa helix aliphatic face. Data in black and grey in both panels correspond to the high and low^32^ P:L ratios. This figure shows that PGLa tilt increases at the high P:L ratio.

The orientation of the PGLa peptide in the bilayer has been studied experimentally using the tilt angle $\gamma$ (see Methods). Because the N- or C-termini exhibit a drop in the helical propensity (Fig. 2), we computed $\gamma$ defined for the peptide region 6-14. (The results do not appreciably change if $\gamma$ is computed for the region 15-20). Figure 4a compares the probability distributions $P(\gamma )$ computed at the high and low P:L ratios. Both distributions are approximately unimodal with the average $\langle \gamma \rangle =107\pm 5^\circ$ and $89\pm 2^\circ$, respectively. Next, we considered the rotation of the aliphatic face of the PGLa helix. To this end, we used the rotation angle $\beta$ (see Methods) defined for Lys19 and computed the probability distribution $P(\beta )$. (Qualitatively similar results have been collected if $\beta$ is defined for Lys15 or Lys12). Figure 4b shows that at the high P:L ratio Lys19 side chain predominantly points up with the average angle $\langle \beta \rangle =105\pm 5^\circ$, and since the peptides are mostly inserted, it is directed up toward the anionic phosphate groups. At the low P:L ratio $\langle \beta \rangle$ is $74\pm 12^\circ$. Thus, changing P:L ratio increases the peptide tilt and maintains Lys19 upward direction. The experimental values for the tilt and Lys12 rotation angles have been reported for the mixed DMPC/DMPG bilayer at 308 K, full hydration, and different P:L ratios^15,16^. At the 1:200 ratio the experimental $\gamma = 98^\circ$ and $\beta =115^\circ$. However, when the P:L ratio increases to 1:20 $\gamma$ and $\beta$ become $121^\circ$ and $113^\circ$.

*Correspondence between simulation and experimental conditions:* To properly compare the simulations and experiments, we need to find the correspondence between the experimental and simulation P:L ratios. Our low P:L ratio simulations, which keep PGLa exclusively monomeric, may roughly correspond to the experimental P:L ratio of 1:200, because our helical fraction $<H>=0.63$ and the PGLa tilt angle $<\gamma >=89^\circ$ agree approximately with the experimental $H=0.60$ and $\gamma =98^\circ$^14,15^. It is more challenging to match our high P:L ratio to the experiment, because by design our simulations do not allow aggregation beyond a dimer. To this end, we computed the quadrupolar splittings $\Delta \nu _q$ (see SI). Based on the direct comparison of in silico and experimental $\Delta \nu _q$ it appears that our high P:L ratio represents the experimental 1:20 P:L ratio (see Table S1)^15^. Incidently, this outcome matches the actual P:L ratio (1:25) in our simulation system. However, some difference in the tilt $\gamma$ and rotation $\beta$ angles between our simulations and experiments must still be noted (see above). These discrepancies can be explained by the differences in angle definitions, difficulties in precise matching experimental and computational conditions, and differences in bilayer composition. Nevertheless, experiments and simulations are in qualitative agreement with respect to data trends, namely, that with the increase in P:L ratio the PGLa helical propensity decreases, while the overall PGLa tilt increases. Simulteneously, the upward direction of Lys side chains in the PGLa helix is preserved.

**Figure 5:**
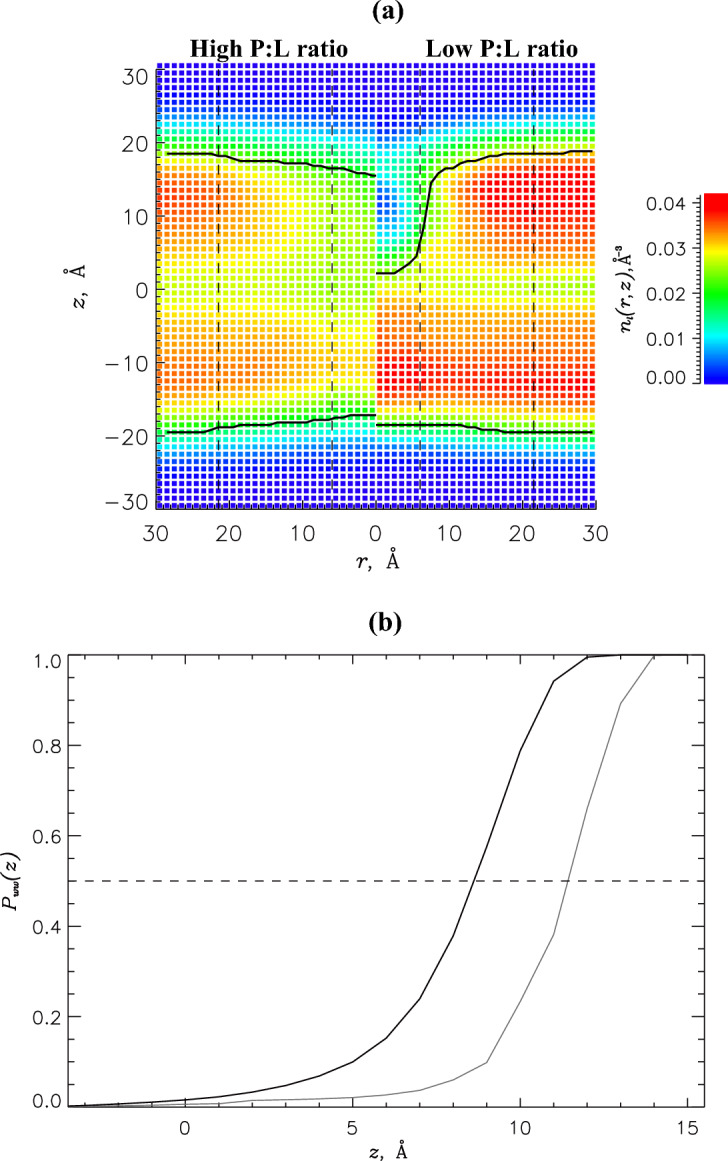
(**a**) The volume number density of lipid heavy atoms $n_l(r,z)$ as a function of the distance *r* to the PGLa center of mass and the distance *z* to the bilayer midplane maps DMPC/DMPG bilayer structure around bound PGLa peptide. Left and right panels compare the density profiles at the high and low^32^ P:L ratios. Continuous black lines mark the bilayer boundaries $z_b(r)$. The boundaries of near, proximal, and distant regions are given by dashed lines. The density distribution $n_l(r,z)$ is computed by permuting the upper and lower leaflets and peptides placing the reference peptide center of mass at $r=0$. This figure implicates surprisingly weak disruption of the bilayer structure at the high P:L ratio. (**b**) The probabilities $P_{ww}(z)$ map the insertion of the water wires to the depth *z* or below. Data for the high and low P:L ratios are in black and grey.

*PGLa impact on the DMPC/DMPG bilayer:* The changes in the DMPC/DMPG bilayer structure caused by PGLa binding were evaluated using the volume number density $n_l(r,z)$ of lipid heavy atoms computed as a function of the distance *r* to the peptide center of mass and the distance *z* to the bilayer midplane (see Methods). Figure 5a compares $n_l(r,z)$ for the high and low P:L ratios. In both cases, PGLa binding creates a lipid density void around the peptide, but its extent depends on the P:L ratio. If the bilayer thinning $\Delta D$ is defined as the difference in the average bilayer thickness *D* in the near and distant regions, then it is $6.3\pm 0.9$ and $17.3\pm 0.3$ Å at the high and low P:L ratios, respectively. The surprising result is that PGLa binding at the high P:L ratio causes much weaker bilayer thinning than at the low. A possible rationale is proposed below after the analysis of surface lipid density.

Our previous study at the low P:L ratio has demonstrated that PGLa induces a water invasion into the DMPC/DMPG bilayer^32^. To check this effect at the high P:L ratio, we computed the probability $P_{ww}(z)$ for a water wire to penetrate the bilayer to the depth *z* or below (see Methods). Figure 5b compares $P_{ww}(z)$ obtained at the high and low P:L ratios. The figure unambiguously show that at the high P:L ratio water invades the bilayer deeper. In fact, at the low P:L ratio half of water wires extends at least to the depth of $z=11.5$ Å compared to $z=8.5$ Å at the high P:L ratio. Thus, the increase in the P:L ratio facilitates water penetration in the bilayer by additional 3 Å. This outcome can be readily understood if we recall that the average position of the PGLa peptide center of mass $z_{CM}$ at the high P:L ratio is 2.5 Å deeper than at the low (Fig. 3a).

In our previous studies at the low P:L ratio, we observed strong interactions between cationic PGLa and anionic DMPG lipids resulting in an influx of these lipids into the peptide proximity with simultaneous efflux of zwitterionic DMPC^31,32^. To examine the same effect at the high P:L ratio, we considered the lipid surface number densities $n_{s,x}$ presented in Table 1. At the high P:L ratio PGLa decreases $n_{s,l}$ in the near region compared to the distant one by merely 16% compared to the two-fold drop at the low ratio. Decomposing densities in Table 1 reveals a negligible increase in the near DMPG surface number density $n_{s,DMPG}$ and a 37% drop in the DMPC density $n_{s,DMPC}$. At the low P:L ratio there is also a minor variation in $n_{s,DMPG}$, but, in contrast, a very sharp, three-fold drop in $n_{s,DMPC}$. Furthermore, at the high P:L ratio the DMPC:DMPG ratio in the near region is 1.2, whereas it is 0.5 at the low P:L. Hence, at the high P:L ratio the efflux of DMPC lipids from the near region, although present, is nevertheless much muted than at the low ratio. In fact, at the high P:L ratio the concentration adjusted DMPG density is still about one-third less than $n_{s,DMPC}$, whereas it surpasses $n_{s,DMPC}$ at the low ratio (Table 1). The plausible reason for this effect is as follows. Table 1 shows that at the low P:L ratio the proximal region exhibits a minor drop in DMPC density compared to the distant region. Then, if at the high ratio the average interpeptide distance is $14.8\pm 0.5$Å, a DMPC lipid occurring in the proximal region of one peptide becomes a “near” lipid for the other. The outcome is a cross-correlation between peptide lipid shells that partially levels off the densities in the near and proximal regions.

The analysis of lipid densities provides an insight into the bilayer thinning. Because at the high P:L ratio the correlations between PGLa lipid shells largely sustain the lipid density near and away from the bound peptides, the bilayer thinning is muted compared to the low P:L ratio. In fact, according to Table 1 the near density $n_{s,l}$ at the high P:L ratio is increased by about 50% compared to that at the low ratio. Furthermore, a weak efflux of DMPC lipids offers a rationale for the reduced helical fraction in PGLa peptides at the high P:L ratio. Because the near bilayer region becomes less anionic, it induces weaker helical propensity in the PGLa peptide due to smaller polarity gradient between the bilayer surface and interior. Partial unraveling of the helix allows lipids to enter the PGLa volume reducing the overall drop in the near lipid density and further alleviating the bilayer thinning. We are not aware of experimental studies probing these effects, apart from the report that PGLa-induced clustering of anionic lipids has been observed in differential scanning calorimetry experiments^34^. Notably, no clustering of DMPG lipids has been detected around another AMP, indolicidin^35^. Although its smaller net positive charge compared to that of PGLa may offer a straightforward reason, another, more subtle contribution cannot be ruled out. Indolicidin experiments were performed at the P:L ratio of up to 1:10, whereas PGLa experiments have used 1:20. Our data argue that high P:L ratio may impede DMPG clustering.

**Table 1:** Lipid densities in bilayer regions.

Bilayer region	$n_{s,l}$,	$n_{s,DMPG}$,	$n_{s,DMPC}$,
	$10^{-2}$Å^-2^	$10^{-2}$Å^-2^	$10^{-2}$Å^-2^
High P:L ratio			
Near	1.22 ± 0.02	0.55 ± 0.06	0.67 ± 0.03
Proximal	1.33 ± 0.04	0.57 ± 0.03	0.76 ± 0.02
Distant	1.46 ± 0.01	0.53 ± 0.01	0.93 ± 0.01
Low P:L ratio^32^			
Near	0.84 ± 0.07	0.56 ± 0.03	0.28 ± 0.05
Proximal	1.53 ± 0.01	0.64 ± 0.02	0.89 ± 0.01
Distant	1.62 ± 0.01	0.58 ± 0.01	1.05 ± 0.01

### Free energy landscape of PGLa binding and dimer formation

**Figure 6:**
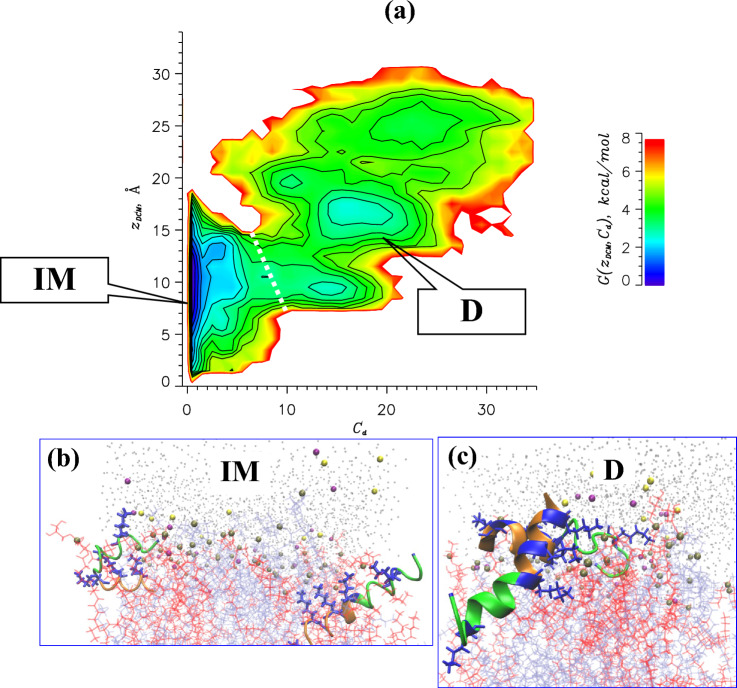
(**a**) The free energy of PGLa peptides in a leaflet $G(z_{DCM},C_d)$ computed as a function of the position of their center of mass $z_{DCM}$ along the bilayer normal and the number of interpeptide contacts $C_d$. The contour lines are incremented by 0.5 kcal/mol. The locations of monomeric **IM** and dimeric **D** species are shown. The dashed white line approximately partitions nascent dimers and “mature” dimers **D**. (**b**) Representative monomer structures are taken from the state **IM**. (**c**) Representative dimer structure is taken from the **D** free energy minimum at $z_{DCM}\sim 17$Å and $C_d\sim 16$. The **D** dimer is positioned at the bilayer-water interface and exhibits a criss-cross interpeptide interface.

According to our previous studies at the low P:L ratio PGLa monomers bind to the DMPC/DMPG bilayer via a two-state mechanism. Specifically, the PGLa peptide adopts a dominant inserted state and a metastable surface bound state^32^. Then, to investigate the free energy of PGLa binding and dimer formation, we selected two reaction coordinates - the position of the center of mass of two PGLa peptides in a leaflet $z_{DCM}$ and the number of interpeptide contacts $C_d$ (see Methods). Figure 6a presents the free energy of PGLa peptides in a leaflet $G(z_{DCM},C_d)$ computed as a function of these two variables. The free energy landscape reveals a thermodynamically stable state, inserted monomers (**IM**), augmented by a manifold of metastable dimeric states. In Fig. 6a one can distinguish the nascent dimers, which are not separated by any free energy barrier from **IM** and represent transient assemblies readily falling apart and converting back to **IM**. To partition nascent from “mature” dimers **D**, we identified the set of saddle points or transition states on the free energy surface $G(z_{DCM},C_d)$ in Fig. 6a. Consequently, we focus our analysis on **IM** and **D**, whose characteristics are listed in Table 2.

**Figure 7:**
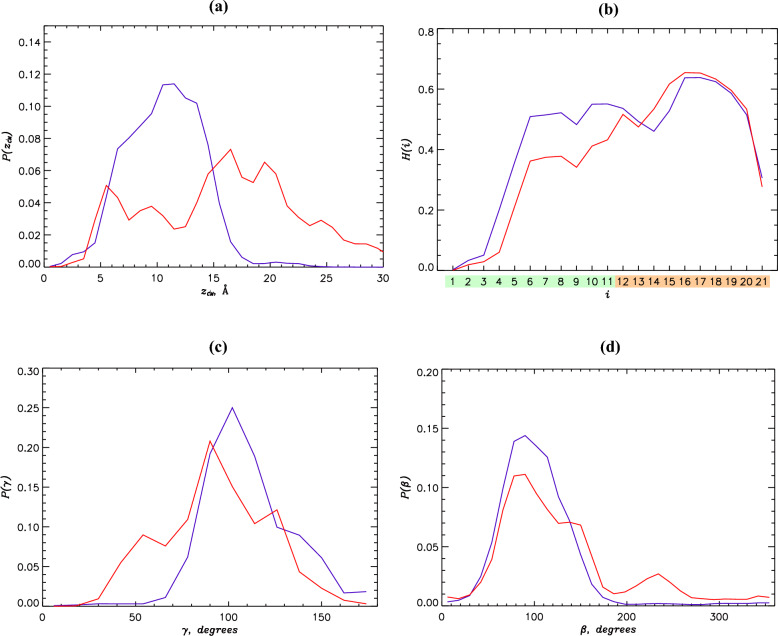
Comparison of PGLa monomers and dimers at the high P:L ratio: (**a**) The probability distributions $P(z_{CM})$ of PGLa peptide center of mass position $z_{CM}$ computed for the monomers **IM** and dimers **D**. (**b**) The helical propensities *H*(*i*) for amino acids *i* are computed for PGLa monomers **IM** and dimers **D**. The amino acids from Nt and Ct regions are colored following Fig. 1a. (**c**) The probability distributions $P(\gamma )$ probe the PGLa peptide tilt within the lipid bilayer. (**d**) The probability distributions $P(\beta )$ describe the orientation of PGLa helix aliphatic face. In all panels data in blue and red represent monomers **IM** and dimers **D**.

According to Table 2 the most thermodynamically stable state, the monomeric **IM**, occurs with the probability $0.71\pm 0.05$ and is displayed in Fig. 6b. It follows from Fig. 7a that **IM** peptides are deeply inserted into the bilayer hydrophobic core positioned, on average, at $z_{CM}= 10.6$ Å from the midplane. **IM** helical fraction is 0.42 and according to Fig. 7b stable helix appears at most positions $i\ge 6$. According to Table 2 the peptide is weakly tilted toward the bilayer core, although Fig. 7c suggests a broad distribution of tilt angles with the standard deviation $\delta \gamma = 19^\circ$. Since **IM** rotation angle $\beta$ in Fig. 7d exhibits a unimodal distribution around the average of 103^∘^, the helix approximately positions its cationic Lys19 side chain upward in the direction of anionic phosphate groups. It is important to note that **IM** differ from the monomeric states observed at the low P:L ratio. The reason originates from the cross-correlation between lipid proximal and near regions surrounding the peptides at the high P:L ratio as described above. Since **IM** species are dominant, the increase in PGLa tilt at the high P:L ratio is primarely due to **IM**. Similarly, the weak bilayer thinning at the high P:L ratio is caused by **IM** species and better mixing of lipids and amino acids at the high P:L ratio.

**Table 2:** PGLa peptide states.

State k	$P(k)$ ^a^	$G(k)$^b^ (kcal/mol)	k$\rightarrow$l^c^	$\Delta G^\dagger$ (kcal/mol_	*H*	$\gamma (k)$ (degrees)	$\beta (k)$ (degrees)	$z_{CM}$ (Å)
IM	0.71 ± 0.05	0.0	IM$\rightarrow$D	3.8	0.42	110	103	10.6
D	0.15 ± 0.04	1.0	D$\rightarrow$IM	1.2	0.39	93	128	16.0

^a^Fraction of PGLa peptides in a state **k**. To find *P*(*k*), we included all structures associated with **k** independent of their $G(z_{DCM},C_d)$.

^b^The free energy of **k** is $$G(k)=-RTlnP(k)$$ provided that *G*(**IM**$$)=0.0$$ kcal/mol.

^c^The transition **k**$\rightarrow$**l** crosses the free energy barrier $\Delta G^\dagger = G^\dagger _{k,min}-G(k)$.

The dimers **D** occur with the probability $0.15\pm 0.04$ and are separated from **IM** by the free energy gap of 1.0 kcal/mol. Importantly, a high free energy barrier of almost 4 kcal/mol occur on the **IM**$\rightarrow$**D** path, whereas a much lower barrier exists along **D**$\rightarrow$**IM** (Table 2). Thus, PGLa **D** dimers once formed can be dissociated into **IM** monomers. The free energy $G(z_{DCM},C_d)$ in Fig. 6a indicates that **D** are diverse species with broad distribution of insertion depths $z_{DCM}$ and the number of interpeptide contacts $C_d$. The representative **D** dimer structure is shown in Fig. 6c. The average position of PGLa center of mass in **D** $z_{CM}$ is 16.0Å, which is more than 5Å above the average location of **IM** peptides (Table 2). Indeed, compared to **IM** the dimer probability distribution $P(z_{CM})$ in Fig. 7a exhibits a striking shift to the bilayer surface. Thus, in contrast to the monomeric species the **D** dimers are partly expelled from the bilayer. It follows from Fig. 7b and Table 2 that apart from a moderate destabilization of helix at the positions 6 through 11 the helical propensity in **D** is generally similar to that of **IM**. According to Fig. 7c the probability distribution $P(\gamma )$ for **D** is broad, but it is still apparent that the **D** peptides have smaller tilt than **IM**. Indeed, according to Table 2 the average **D** tilt $\gamma$ is $93^\circ$ whereas it is $110^\circ$ for **IM**. Furthermore, the probability distributions of rotation angles $P(\beta )$ in Fig. 7d and the average $\beta$ for **D** and **IM** are also distinct. Although **D** $P(\beta )$ is broad, it is extended toward larger values compared to **IM**. As a result Lys19 side chain direction deviates, on an average, by almost 40^∘^ (50^∘^ for Lys12) from the bilayer normal, whereas in **IM** the respective deviation is merely 13^∘^. In summary, there are three differences between **IM** and **D**. First, **D** dimers are partially expelled from the DMPC/DMPG bilayer residing near the lipid phosphate groups. Second, the **D** peptides tend to align parallel to the bilayer surface. Third, **D** Lys side chains are slanted away from the bilayer normal. It is of note that because of low stability of **D** the simulation quadrupolar splittings represent the mixture of **IM** and **D** states.

Next, we examine the dimer interface. The average number of interpeptide contacts in **D** is 12.8, the most probable of which are listed in Table S2. It is noteworthy that out of 18 contacts occurring with the probability $P_c>0.1$, only one (and the least stable) involve Lys amino acid, whereas all others are established between hydrophobic amino acids. In fact, in descending order PGLa dimer formation most frequently involves Ala17, Ala20, Ile13, and Leu21. Furthermore, out of 18 contacts 13 or 72% occur between C-terminal amino acids, but most contacts are off-registry. To determine the **D** dimer interface geometry, we computed the angle between the peptides $\alpha$ (see Methods). The probability distribution $P(\alpha )$ in Fig. S5 shows a sharp peak at $\alpha \simeq 100^\circ$ resulting in the average value of 93^∘^. Thus, although $P(\alpha )$ in Fig. S5 implicates a multitude of peptide alignments in **D**, overall the dimers prefer an approximately criss-crossed off-registry interface.

The analysis of dimer interface shed light on the differences between **IM** and **D**. Because PGLa dimer formation almost exclusively involves apolar amino acids positioned on the opposite face of PGLa helix from the Lys side chains, the latter ones tend to point outward in the dimer. Consequently, they could not be directed toward the phosphate groups in both peptides if inserted deeply into the bilayer. Our previous studies have implicated the electrostatic interactions between Lys and lipid phosphate groups as a major factor in PGLa binding^31,32^. Then, to maximize the Lys interactions with lipid phosphate groups while preserving the dimer interface, **D** must position itself close to the bilayer surface. Another reason for the dimer expulsion is that dimer assembly in the bilayer hydrophobic core brings Lys close in a low dielectric environment. Therefore, to minimize electrostatic repulsion while preserving the dimer, it should be repositioned closer to water, which provides better electrostatic screening. The PGLa peptides in **D** state comply with these scenarios by shifting closer to the bilayer surface and slanting Lys side chains away from the bilayer normal.

Previous simulations have investigated PGLa dimers. In particular, Ulmschneider et al. have performed constant temperature simulations probing the PGLa monomers and dimers^29^. The study reported an increase in the PGLa dimer tilt angle $\gamma$ to 104-107^∘^, which is almost identical to our $\langle \gamma \rangle$. However, upon closer examination this agreement is entirely attributed to the dominant monomeric state **IM** in Table 2, whereas our PGLa dimers **D** exhibit, on an average, no tilt. It is of note that our simulations study de novo PGLa dimer assembly using enhanced sampling, whereas previous studies examined the stability of preconstructed dimers. Furthermore, previous simulations suggested that antiparallel dimer interface is the most stable supported by hydrophobic contacts between Leu, Ala, Gly side chains. In contrast, we observed criss-crossed dimer interface geometry and saw no significant contribution from Gly to the dimer interface. This outcome is interesting, because multiple experimental methods pointed to the critical contribution of Gly7, Lys15, and Lys19 to PGLa-magainin heterodimer assembly^20^. A similar conclusion on the importance of salt bridges has been made in the small angle X-ray and neutron scattering experiments^21^. Therefore, our study, which did not find these interactions to contribute to dimer formation, implicates very different interfaces in PGLa homodimer and PGLa-magainin heterodimer. Finally, we can compare the free energies of dimer formation extracted from our simulations and oriented circular dichroism experiments^19^. The probability of PGLa monomers $P_m$ in our simulations is $0.71\pm 0.05$, whereas the probability of forming dimers, including mature **D** and nascent in Fig. 6a, is $P_d=1-P_m=0.29\pm 0.05$. Then, assuming a simple monomer-dimer equilibrium, one can compute the free energy of dimer formation as

1 $$\begin{aligned} \Delta G_d = -RTln(c_0[D]/[M]^2) = -RTln(c_0VP_d/P_m^2) = \text {-2.4 kcal/mol}, \end{aligned}$$

where $V=$59 Åx 59 Åx 37 Å is the simulation volume available for the pair of peptides and $c_0=1$ M is the standard concentration. This estimate is consistent with the experimental value of $-2.4$ kcal/mol reported for the DMPC bilayer^19^. The agreement should be considered approximate, because our study used anionic DMPC/DMPG bilayer, and Eq. (1) strictly holds only for large simulation systems^36^. Nevertheless, both simulations and experiments agree that the dimerization propensity of PGLa peptides is weak, and association of PGLa with magainin is required for synergistic antimicrobial activity. Poor optimization of PGLa for forming homodimers may indicate that this peptide perform additional biological functions beyond that of AMP.

## Models and methods

### Simulation system

The simulation system consisted of the DMPC/DMPG (3:2) lipid bilayer formed by 38 anionic DMPG and 60 zwitterionic DMPC lipids, four PGLa peptides, two of which were placed on either bilayer side, and 4410 water molecules (Fig. 1). The system net charge was set to zero by adding 38 sodium ions and 20 chlorine ions. The dimensions of the system were approximately 59 Å x 59 Å x 74 Å. Thus, the P:L ratio is about 1:25. The DMPC/DMPG bilayer was selected because it is well studied experimentally^37^ and frequently used in the PGLa experiments. The simulation system is symmetric, in which two PGLa monomers bind and dimerize in either bilayer leaflet effectively doubling the conformational sampling. Such design also reduces the pressure disparity in the leaflets^38^. PGLa peptides were modeled using the all-atom CHARMM22 force field with CMAP corrections^39^, whereas the all-atom CHARMM36 force field was used for lipids^40^ (see SI). A modified TIP3P model was utilized for water^41,42^. To mimic experiments, the PGLa C-terminus was amidated, while the N-terminus was protonated and positively charged resulting in the PGLa net charge of +5^7^.

### Replica exchange simulations

Following our previous studies we used isobaric-isothermal replica exchange molecular dynamics with solute tempering (REST) to probe binding of PGLa to the lipid bilayer^43^. All details of REST implementation can be found in our previous publication^32^. In brief, we considered $R=16$ system replicas, which were simulated at the temperatures distributed exponentially from $T_0=330$ to $T_{R-1}=450$ K. REST interaction scaling effectively divides the system into tempered (“hot”) and untempered (“cold”) partitions. The PGLa peptides along with chloride counterions constituted the “hot” solute, whereas the DMPC/DMPG bilayer, water, and remaining ions were “cold”. By restricting tempering to the solute, REST considerably reduces the computational load compared to traditional replica exchange. However, “cold” solute may slow down system equilibration requiring to extend REST simulations or elevate sampling temperature^32^. A possible solution suggested recently by us is to use replica exchange with hybrid tempering (REHT)^32^. However, because REHT requires an increase in *R*, we opted to use REST, but elevated sampling temperature to compensate for REST deficiencies. Replica exchanges were attempted every 2 ps.

NAMD with REST implementation^44^ along with in-house scripts were used to manage the REST simulations. We used periodic boundaries and 1 fs integration timestep. Hydrogen-associated covalent bonds were constrained by SHAKE. To compute electrostatic interactions we applied Ewald summation, whereas force switching was utilized to turn off van der Waals interactions in the interval from 8 to 12 Å. Temperature was controlled by underdamped Langevin dynamics with a damping coefficient $\gamma =5$ $ps^{-1}$. To maintain pressure of 1 atm the Nosé-Hoover Langevin piston method was applied with a piston period and decay of 200 and 100 fs, respectively. Because the system includes the lipid bilayer, a semi-isotropic pressure coupling was used, which couples *x*, *y* dimensions but adjusts *z* independently. Following our previous studies^45^ the structural integrity of the bilayer at all REST temperatures was preserved by restraining the center of mass of phosphorus atoms in each leaflet with soft harmonic potentials to its average distance from the bilayer midplane $z_P=17.56$ Å at 330 K^31^. Separate set of soft harmonic restraints acted in the *z* dimension to prevent the aggregation of PGLa peptides across periodic boundaries. As designed these restraints do not prevent formation of cross-leaflet aggregates. The impact of these restraints has been carefully evaluated previously and found negligible^46^.

The random initial conditions for REST simulations were prepared as follows. Previous REHT simulations of PGLa monomers have found that the peptide samples the predominant inserted and metastable surface bound states^32^. Accordingly, we selected equal number of inserted and surface bound structures and combined them randomly creating 30 inserted, 30 surface bound, and 36 mixed pairs of peptides. In total, we used 96 distinct pairs of PGLa peptides to initiate high P:L ratio simulations. The initial structures for upper and lower leaflets were always distinct. Importantly, in the initial structures few peptides formed interpeptide interactions. Three independent REST trajectories each of 400 ns were produced. We monitored multiple quantities for evidence of equilibration (see SI). Taken together, REST simulations collected 1.2 μ of sampling at 330 K, of which 0.24 μs was considered equilibrated (0.48 μs per peptide pair).

### Computation of structural probes

Multiple structural quantities were employed to describe PGLa structures, their dimerization, and interactions with the bilayer. PGLa secondary structure was determined using the program STRIDE^47^. An amino acid was considered helical if STRIDE assigned it to $\alpha$-, $\hbox {3}_{{10}}$-, or $\pi$-helix. Then, a helical propensity *H* is the probability for an amino acid to be in a helical state. Similar definitions were applied to other secondary structure types. To map contacts between amino acids, the centers of mass of their side chains were computed (Fig. 1). If their centers of mass were separated by less than 6.5 Å, a contact was formed. The 6.5 Å cut-off approximately corresponds to the onset of hydration of side chains as their separation grows. To probe the formation of PGLa dimers, we defined the number of side chain contacts $C_d$ between the peptides in a leaflet.

To describe the positions of amino acids within the bilayer we computed the probability *P*(*z*; *i*) for an amino acid *i* to occur at a distance *z* from the bilayer midplane. An amino acid *i* is inserted into the bilayer if $z(i)<z_P=17.56$ Å, where *z*(*i*) is its center of mass position along the bilayer normal and $z_P$ is the average position of phosphorus atoms center of mass in a leaflet in the peptide-free bilayer at 330 K^31^. To map the bilayer disruption caused by PGLa, we defined the volume number density of bilayer heavy atoms, $n_l(r,z)$, computed as a function of the distance *r* to the peptide center of mass and the distance *z* to the bilayer midplane. Similar distribution $n_w(r,z)$ was obtained for water. With $n_l(r,z)$ and $n_w(r,z)$ computed the bilayer boundary $z_b(r)$ and the bilayer thickness *D* can be determined as described^46^. (Note that $z_b(r)$ solely monitors the drop in the bilayer atom density.) Surface number densities of all lipids, $n_{s,l}$, or $x=$DMPC and DMPG lipids, $n_{s,x}$, were computed. To this end, lipids were represented by their phosphorus atoms. To compare the bilayer properties near the peptides and in the peptide-free region, we classified lipids into near, which occur within the distance $r < 6$ Å from the PGLa peptide center of mass, proximal with 6 Å$<r<21.5$ Å, and distant with $r>21.5$ Å. The diameter of the near region corresponds well to the bound PGLa average radius of gyration $\langle R_g \rangle =12$ Å^31^. The boundary of a distant region corresponds to the distance *r* from the peptide, where $z_b(r)$ reaches constant. (Such isotropic in *xy* plane, averaged definitions of the bilayer regions may not always be perfect in partitioning lipids into three classes. However, as long as they are applied consistenly at the low and high P:L ratios, they still detect differences in lipid distributions.)

To find water wires, we considered all water molecules occurring below the average position of the center of mass of phosphorus atoms in a leaflet $z_P$. A water wire is then a chain of hydrogen bonded water molecules extended from the bulk water above $z_P$. The position of the water oxygen with the smallest *z* in the wire represents its depth. To define a hydrogen bond between the donor D and acceptor A atoms, we assumed that the distance between them must be less than 4 Å and the angle $\angle DHA > 150^\circ$.

The tilt angle $\gamma$ represents the angle between a peptide region and the bilayer normal. Following Reischer et al.^28^ we divided a peptide region in two halves and computed their centers of mass. The angle between the vector $\textbf{h}$ connecting their centers of mass and the bilayer normal is $\gamma$. If $\gamma = 90^\circ$, a peptide region is parallel to the bilayer. Also following Reischer et al.^28^ we defined the rotation angle $\beta$, which characterizes the orientation of a residue in a helix. To this end, the coordinate system was translated and rotated to align the helix peptide region vector $\textbf{h}$ along the x-axis, while keeping y-axis parallel to the bilayer surface. Then, $\beta$ for amino acid *i* is the angle between the vector connecting the helix axis and the C_α_ atom of *i* and the y-axis. If so, $\beta =90^\circ$ or 270^∘^ correspond to amino acid *i* pointing up or down along the bilayer normal. The definition of $\beta$ is unambiguous only if a peptide region adopts a helix positioned approximately within the bilayer plane. To probe the orientation of peptides in the dimer, we computed the angle $\alpha$ between the vectors $\textbf{h}$ defined for the PGLa region Lys15 to Ala20. These amino acids were selected because PGLa dimer assembly mostly involves C-termini.

All structural quantities are computed using the weighted-histogram analysis method^48^ at 330 K and represent thermodynamic averages denoted with angular brackets $\langle ... \rangle$. Standard errors were determined by treating each REST trajectory as independent sample. Then, the total number of samples is $n=3$. Due to high P:L ratio we screened all conformational snapshots for the interactions of PGLa monomers or dimers with their periodic images. We found that 8.5% of structures contained such artifacts and accordingly those were eliminated from the conformational analysis. Our tests showed that, because these spurious interactions affect a small fraction of all conformations, their elimination does not appreciably change the PGLa properties.

### Supplementary Information

Supplementary Information.

## Data Availability

The datasets generated and analyzed during the current study are available from the corresponding author on reasonable request.
