# Supplementary material for: Binding and dimerization of PGLa peptides in anionic lipid bilayer studied by replica exchange molecular dynamics
Source: Sci Rep. 2024 Feb 29;14:4972. doi: 10.1038/s41598-024-55270-8 (PMC10904749; doi:10.1038/s41598-024-55270-8)
Supplement: Supplementary file 1 — Supplementary Information. [file 41598_2024_55270_MOESM1_ESM.pdf]

Supplementary Information

Binding and dimerization of PGLa peptides in anionic lipid bilayer studied by replica exchange molecular dynamics

Steven R. Bowers, Christopher Lockhart, and Dmitri K. Klimov
School of Systems Biology, George Mason University, Manassas, VA 20110
E-mail: dklimov@gmu.edu

Selection of peptide force field: For PGLa peptides we have selected the all-atom CHARMM22 force field with CMAP corrections [1]. As described previously [2], we have tested a more recent CHARMM36m force field [3] but found that PGLa peptide does not adopt helical state upon binding to the DMPC/DMPG bilayer, which was observed experimentally [4]. Recent study of the antimicrobial peptide melittin has reached the same conclusion [5].

Performance of REST algorithm: We employed replica exchange with solute tempering (REST) molecular dynamics to sample the conformational space of PGLa peptides binding to the DMPC/DMPG bilayer at the high peptide:lipid (P:L) ratio. Our REST simulations utilized R=16 temperature conditions exponentially distributed from 330 to 450 K (see Methods). Properly set up REST simulations generate random replica walks across temperatures. Fig. S1 visualizes replicas walking over REST temperatures in a REST trajectory. The color mosaic in this figure suggests that replicas are not trapped at any REST temperature for a protracted period.

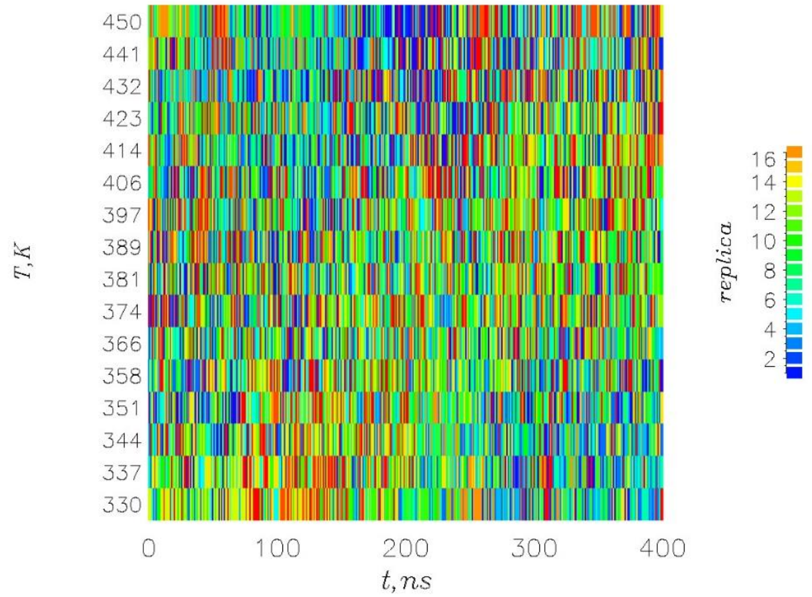

Figure S1 A walk of replicas over REST temperatures in a representative REST trajectory. A color scale codes the allocation of replicas over temperatures at the beginning of trajectory. Similar behavior was seen in the other two REST trajectories.

Numerically, the extent of random distribution of replicas over temperatures is measured using replica mixing parameter [6]

$$m(T) = 1 - \frac{\sqrt{\sum_{r=0}^{R-1} t_r^2}}{\sum_{r=0}^{R-1} t_r}, \quad \text{(S1)}$$

where T is the REST temperature and t_r is the total number of REST iterations spent by replica r at T. Assuming fully random mixing of R=16 replicas over temperatures, the theoretical maximum value is $m = 1 - 1/R^{1/2} = 0.75$. Fig. S2a reveals that $m(T)$ approaches this theoretical maximum at most REST temperatures, particularly in the middle of temperature range. The average value of m is 0.68. Fig. S2b shows another measure of REST performance, the replica exchange rate $\alpha(T)$, which, if averaged over all REST temperatures, is 0.28. The observed range of $\alpha(T)$ is within the optimum for replica exchange simulations [7]. Thus, Figs. S1 and S2 support efficient replica mixing across temperatures, which is a prerequisite for equilibrium REST sampling.

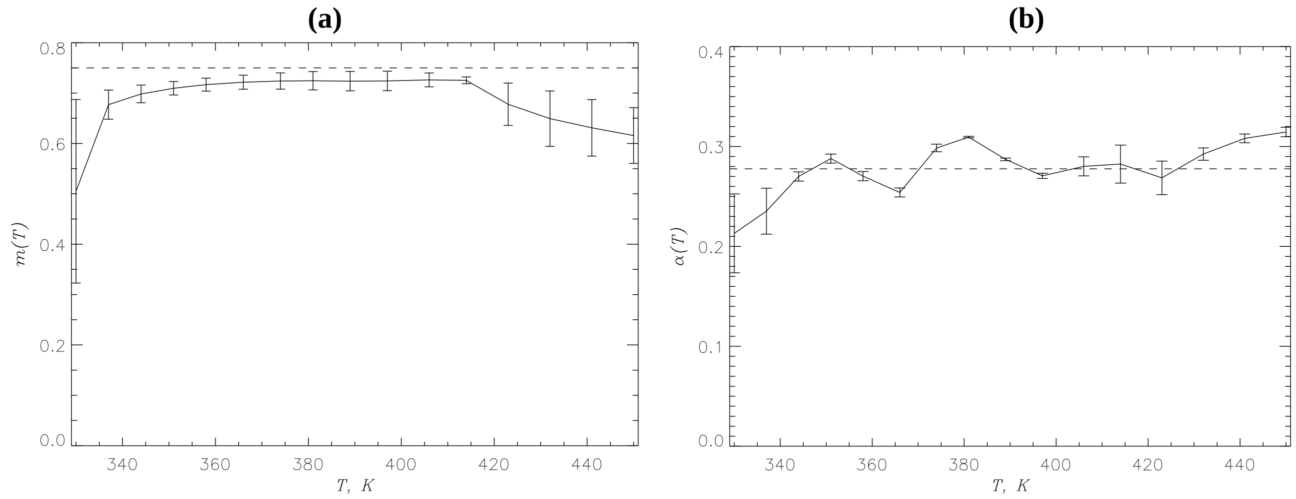

Figure S2 (a) The replica mixing parameter $m(T)$ computed for all REST temperatures T. Dashed line indicates a theoretical maximum for $m(T)$. (b) The replica exchange rate $\alpha(T)$ computed for REST temperatures T. The dashed line shows the average α over all temperatures. The data in both panels is averaged over three REST trajectories. Vertical bars represent standard errors.

Convergence of REST sampling: To check that the PGLa peptides binding the DMPC/DMPG bilayer approach equilibrium, we used three quantities. First, to evaluate the convergence of PGLa secondary structure sampling, we computed the PGLa helical fraction, $H(t)$, as a function of REST time t at 330 K. To investigate the equilibration of PGLa in the bilayer, we computed the position of the center of mass of PGLa peptide, $z_{cm}(t)$. Finally, to probe the PGLa dimer formation, we examined the number of interpeptide contacts $C_d(t)$. All three quantities presented in Fig. S3 approached approximate baselines after 320 ns indicating that the last 80 ns of sampling in each trajectory can be treated as equilibrated. As with any simulation we cannot rule out that its extension would not reach new free energy minima, but the available sampling data give no evidence to that extent. Thus, we tentatively assumed that the simulations converged.

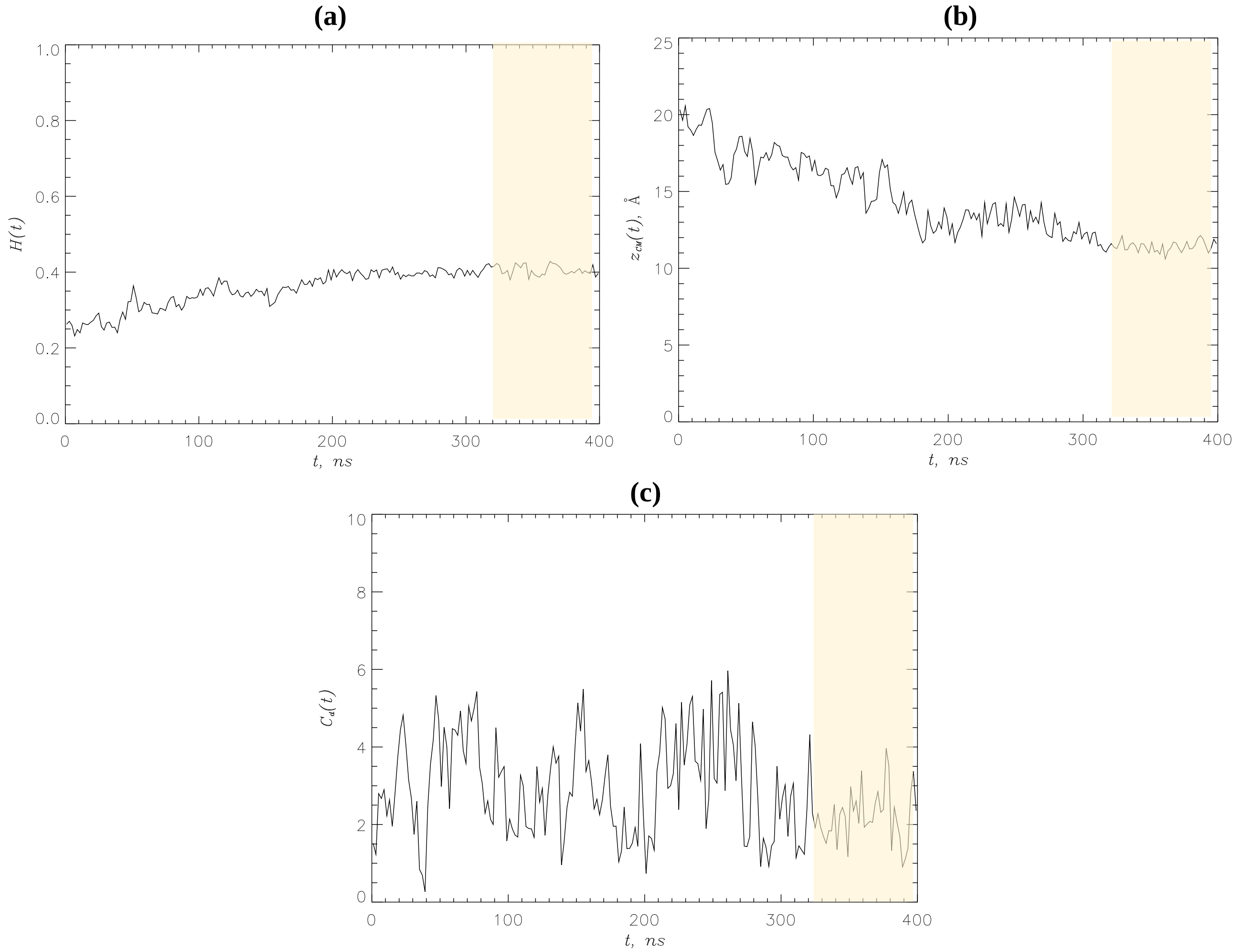

Figure S3 PGLa helical propensity $H(t)$, the position of PGLa center of mass $z_{cm}(t)$, and the number of interpeptide contacts $C_d(t)$ are shown in panels (a-c), respectively, as a function of REST time t. The data in (a-c) are averaged over the three REST trajectories and collected at 330 K. The shaded region at t > 320 ns represents the onset of equilibration. Panels (a-c) suggest sampling convergence in our REST simulations.

Temperature dependence of PGLa properties: Our initial attempts to use REST simulations and reach convergence of PGLa binding and dimer formation at 310K have failed. Consequently, to improve convergence PGLa sampling has been performed at slightly elevated temperature of 330K. Because as a result we compare PGLa properties at the high and low P:L ratios using the sampling at 330 and 310 K, it is important to rule out temperature impact on PGLa properties. In principle, experimental data indicate that PGLa orientation in the DMPC/DMPG bilayer is temperature dependent as the peptide tilt is reduced from γ = 130° in the temperature range from 278 to 308 K to 96° at 318 K [8]. However, CHARMM force field does not reproduce well the temperature induced changes in PGLa tilt. Indeed, Fig. S4 presents the average positions of amino acid centers of mass $\langle z(i)\rangle$ computed at the low P:L ratio and two temperatures, 310 and 330 K. It is seen that the PGLa monomer insertion remains virtually unchanged despite 20 K temperature difference. Furthermore, the average tilt angle of the peptide region 6-14 $\langle\gamma\rangle$ is 94±2° and 97±5°

at 310 and 330 K, respectively. Given the sampling error, ⟨γ⟩ shows no apparent temperature dependence. These outcomes are not unexpected and consistent with previous studies, which showed that the temperature dependence of peptide dimensions in CHARMM36 force field in a wide temperature range from 283 to 323 K is weak [9].

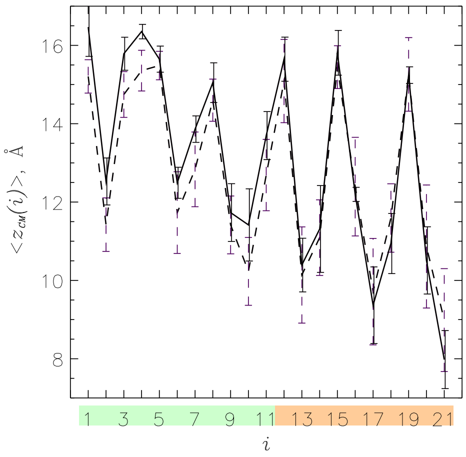

Figure S4 The average positions of PGLa amino acid centers of mass ⟨$z(i)$⟩ computed at two temperatures, 310 K (continuous line) and 330 K (dashed line). The data were collected using replica exchange with hybrid tempering simulations and computed via weighted histogram method [10]. The amino acids from Nt and Ct regions are colored following Fig. 1a.

Comparison of *in silico* and experimental quadrupolar splittings: To provide direct test of our simulations against experimental data, we computed quadrupolar splittings $\Delta\nu_q$ using the following equation [11]

$$\Delta\nu_q = K\langle 3cos^2\vartheta - 1\rangle, \quad \text{(S2)}$$

where θ is the angle between the Cα-Cβ bond and the bilayer normal and K is a constant. The splittings were computed directly from REST sampling without an assumption of PGLa helical structure. We computed $\Delta\nu_q$ for amino acids i=6,8,10,14, because for these positions experimental $\Delta\nu_q$ are available for the DMPC/DMPG (3:1) bilayer at the P:L ratios of 1:200, 1:50, and 1:20 [12]. The results shown in Table S1 suggest excellent correlation between our and experimental $\Delta\nu_q$ at 1:20 P:L ratio, which is reduced at 1:50 and absent at 1:200.

Table S1 *In silico* and experimental quadrupolar splittings $\Delta\nu_q$ and their comparison

**PGLa residue**	**REST simulations**[a]	**MLV samples**[b]		**Oriented samples**[b]	
		**1:200**[c]	**1:50**[c]	**1:50**[c]	**1:20**[c]
6	0.09	14.4	12	17.5	12.2
8	0.36	18.4	36	39.2	35.7
10	0.33	15	20	27.5	26.7
14	0.28	25.6	16.6	20	18.6
Correlation coefficient[d]		0.34	0.77	0.77	0.88

[a] $\Delta\nu_q$ are computed assuming arbitrary K and represent absolute values of quadrupolar splittings
[b] data in kHz from [12]
[c] experimental P:L ratio
[d] correlation between simulation and experimental $\Delta\nu_q$

PGLa dimer interface: To examine the interactions responsible for the assembly of PGLa dimers, we computed the probabilities $P_c(i,j)$ of forming contacts between amino acids i and j from different peptides in a leaflet. Table S2 presents the list of interpeptide contacts in the **D** interface with $P_c > 0.1$. To examine the alignment of peptides in **D**, we computed the angle α between them (see Methods). The probability distribution $P(\alpha)$ is presented in Fig. S5. The results are discussed in the main text.

Table S2 The list of interpeptide interactions in the **D** dimers.

**Amino acid *i***	**Amino acid *j***	**Probability $P_c(i,j)$**
Ala17	Leu21	0.22
Leu18	Leu21	0.19
Val16	Ala17	0.17
Ile13	Ala17	0.17
Ala17	Ala17	0.17
Ile9	Ala20	0.17
Ile13	Ala20	0.15
Ala14	Ala20	0.15
Ala17	Ala20	0.15
Leu18	Ala20	0.14
Ala14	Leu21	0.14
Ile13	Leu21	0.14
Ala8	Val16	0.14
Ile13	Val16	0.13
Ala17	Leu18	0.13
Ile9	Lys19	0.11
Ala8	Ala20	0.10
Ile9	Ala17	0.10

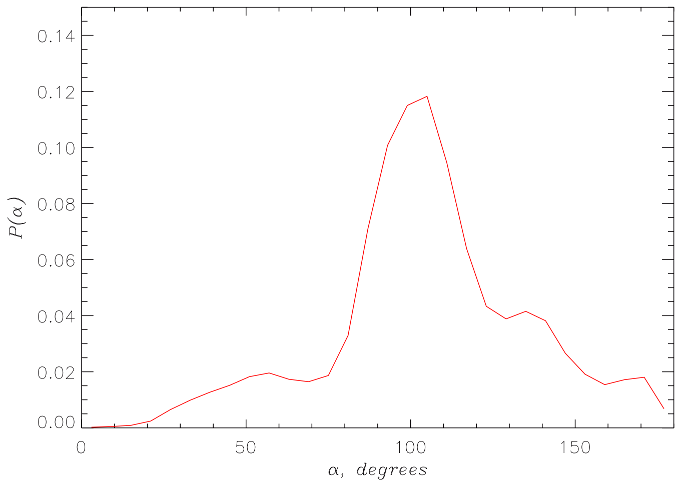

Figure S5 The probability distribution $P(\alpha)$ probes the alignment of peptides in the **D** dimers.

References:

[1] Buck, M., Bouguet-Bonnet, S., Pastor, R. W., & MacKerell, Jr., A. D. (2005) Importance of the CMAP correction to the CHARMM22 protein force field: Dynamics of hen lysozyme. *Biophys. J.* **90**, L36-L38.

[2] Bowers, S., Klimov, D. K., & Lockhart, C. (2022) Mechanisms of binding of antimicrobial peptide PGLa to DMPC/DMPG membrane. *J. Chem. Inf. Model.* **62**, 1525-1537.

[3] Huang, J., Rauscher, S., Nawrocki, G., Ran, T., Feig, M., de Groot, B. L., Grubmüller, H., & MacKerell, Jr., A. D. (2016) CHARMM36m: An improved force field for folded and intrinsically disordered proteins. *Nat. Methods* **14**, 71-73.

[4] Bechinger, B., Zasloff, M., & Opella, S. J. (1998) Structure and dynamics of the antibiotic peptide PGLa in membranes by solution and solid-state nuclear magnetic resonance spectroscopy. *Biophys. J.* **74**, 981–987.

[5] Fox, S. J., Lakshminarayanan, R., Beuerman, R. W., Li, J., & Verma, C. S. (2018) Conformational transitions of melittin between aqueous and lipid phases: Comparison of simulations with experiments. *J. Phys. Chem. B* **122**, 8698-8705.

[6] Han, M. & Hansmann, U. H. E. (2011) Replica exchange molecular dynamics of the thermodynamics of fibril growth of Alzheimer's Aβ42 peptide. *J. Chem. Phys.* **135**, 065101.

[7] Denschlag, R., Lingenheil, M., and Tavan, P. (2009) Optimal temperature ladders in replica exchange simulations. *Chem. Phys. Lett.* **473**, 193-195.

[8] Afonin, S., Grage, S. L., Ieronimo, M., Wadhwani, P., & Ulrich, A. S. (2008) Temperature-dependent transmembrane insertion of the amphiphilic peptide PGLa in lipid bilayers observed by solid state 19F NMR spectroscopy. *J. Amer. Chem. Soc.* **130**, 16512–16514.

[9] Jephthah, S., Staby, L., Kragelund, B. B., & Skepo, M. (2019) Temperature dependence of intrinsically disordered proteins in simulations: What are we missing? *J. Chem. Theor. Comput.* **15**, 2672-2683.

[10] Bowers, S. R., Lockhart, C., & Klimov, D. K. (2023) Replica exchange with hybrid tempering efficiently samples PGLa peptide binding to anionic bilayer. *J. Chem. Theor. Comput.* **19**, 6532–6550.

[11] Reiser, S., Strandberg, E., Steinbrecher, T., Elstner, M. & Ulrich, A. S. (2018) Best of two worlds? How MD simulations of amphiphilic helical peptides in membranes can complement data from oriented solid-state NMR. *J. Chem. Theory Comput*. **14**, 6002–6014.

[12] Tremouilhac, P., Strandberg, E., Wadhwani, P. & Ulrich, A. S. (2006) Conditions affecting the re-alignment of the antimicrobial peptide PGLa in membranes as monitored by solid state 2H-NMR. *Biochim. Biophys. Acta* **1758**, 1330–1342.
